# Robotic animals as new tools in rodent neuroscience research: proposed applications of zooinspired robots for mouse behavioral testing

**DOI:** 10.3389/fnbeh.2025.1545352

**Published:** 2025-02-24

**Authors:** Raffaele d’Isa

**Affiliations:** Institute of Experimental Neurology (INSPE), Division of Neuroscience (DNS), IRCCS San Raffaele Scientific Institute, Milan, Italy

**Keywords:** biorobotics, ethorobotics, animal robot, robotic animal, robot-animal interactions, animal welfare, mice

## Abstract

Bioinspired robots are machines which reproduce structural or functional features of a living organism. In particular, the bioinspired robots which reproduce features of animals can be more specifically defined as zooinspired robots. Currently, the applications of animal robots are various and range across different fields, such as, for instance, nature conservation, search and rescue of humans after natural or man-made disasters, exploration of extraterrestrial environments and robotic pets for elderly people under care for dementia. Several animal species have been imitated up to now, from lizards to butterflies, and from fish to dogs. Animal robots used to investigate the social behavior of an animal species through animal-robot interactions are called ethorobots. Intriguingly, ethorobots are able to reproduce in the laboratory behaviors that are generally produced spontaneously in nature and are difficult or impossible to evoke and modulate in captive animals, which makes these animal robots particularly useful tools for experimental ethology and ethological neuroscience. Rodents, primarily mice and rats, are the most common animal model in biomedical research. Coherently with the importance of these species for scientific research, robotic mice and rats have been attracting increasing efforts in bioinspired robotics over the course of the past five decades. The technological advancement of animal robots will make their employment for scientific research increasingly useful. However, clear experimental applications of animal robots should be identified in order to challenge engineers to design robots that can serve these experimental scopes. In the present work, we will describe possible practical applications of robotic animals for mouse behavioral testing across six different behavioral domains, namely courtship, parental care, antipredatory behavior, helping behavior, predation and territory defense-related aggression. In particular, we will outline how robotic animals could be employed to interact with living mice in a series of specific tests of social behavior. Finally, in the conclusion we will consider the ethical and epistemological advantages of the use of robotic animals in behavioral neuroscience. Indeed, robotic animals can benefit scientific research on social behaviors both in terms of optimized animal welfare of the tested subjects and of extended opportunities of experimental designing due to an unprecedented control over the independent variables.

## 1 Introduction

Biomimetic and bioinspired robots are machines designed to reproduce structural or functional features of biological organisms. In particular, biomimetic robots are robots which reproduce a feature of the structure or the function of a living organism by copying directly the structure or function of the imitated organism (Meyer and Guillot, 2008). On the other hand, bioinspired robots are robots which reproduce a feature of the structure or the function of a living organism, but through a type of structure or mechanism actually not possessed by the imitated organism (Meyer and Guillot, 2008). Biomimetics and bioinspiration can be considered as part of a continuum, in which both employ an idea deriving from the observation of a biological organism, but at a different degree. Indeed, while the first copies faithfully the idea, the latter only uses it as inspiration. Consequently, while for bioinspired robots the similarity with the imitated organism may be loose, for biomimetic robots the similarity with the imitated biological organism is generally stronger.

Biomimetics and bioinspiration underwent a great development over the course of past 25 years, to the point that biomimesis has been defined as the “new wave” of robotics (Paulson, 2004; Meyer and Guillot, 2008) and bioinspiration as “something for everyone” (Whitesides, 2015). In 2006 a specialized journal devoted to the field was founded: Bioinspiration & Biomimetics (ISSN: 1748-3190).

More specifically, bioinspired robots reproducing features of animals can be defined as zooinspired robots. Zooinspired robots have a long history. For instance, in 1509 the Italian inventor Leonardo da Vinci (1452–1519) designed and built a robotic lion that was capable of automated locomotion, a mechanical automaton which was used to celebrate the entry of King of France Louis XII (1462–1515) in Milan in 1509 and the entry of King of France Francis I (1494–1547) in Lyon in 1515 (Vasari, 1550; Rosheim, 2001; Burke, 2006).

The applications of animal robots are diverse and range across different fields, including, for example, nature conservation (Chellapurath et al., 2023), search and rescue of humans after natural or man-made disasters (Wei and Ge, 2014), exploration of extraterrestrial environments (Chen G. et al., 2023) and robotic pets for elderly people under care for dementia (Petersen et al., 2017). Considering nature conservation alone, bioinspired robots have been proposed for five roles: exploration, data collection, monitoring, intervention and ecosystem maintenance (Chellapurath et al., 2023). Animal robots can also be used for educational purposes, for instance in academic courses of comparative psychology (Brahmandam et al., 2020) or to bring the general audience closer to technological design and increase the public engagement with science (Siddall et al., 2023).

Several animal species have been imitated to create robotic animals, for instance, only to mention some of the most recent achievements: an octopus arm (capable of reaching, sensing and grasping) which can be remotely controlled through a special glove wearable on a single finger (Xie et al., 2023; Nogrady, 2023), a robotic lizard for Mars surface exploration (Chen G. et al., 2023), a robotic water strider capable of free-floating in the gravity-free environment of space (Sai et al., 2023), a robotic gecko capable of stable motion in the microgravity environment of space stations (Pei et al., 2023), a soft robotic dog capable of standing and fast trotting (Li et al., 2023), a robotic butterfly that can swim underwater driven by light (Guo et al., 2023), a robotic sea snake for underwater infrastructure inspection or search-and-rescue operations in sunk vessels (Bianchi et al., 2024) and a biomimetic robotic fish that can monitor the quality of water in aquacultures (Chen X. et al., 2023).

Animal robots can also be important in scientific research, especially in experiments studying the social behavior of living animals (Webb, 2000, 2008; Patricelli, 2010; Krause et al., 2011; Halloy et al., 2013; Mitri et al., 2013; Frohnwieser et al., 2016; Hughey et al., 2018; Abdai et al., 2018; Romano et al., 2019, 2024; Romano and Stefanini, 2021; Landgraf et al., 2021; Papaspyros et al., 2024). The research field employing robots to study and/or modulate animal behavior has been defined as ethorobotics (Datteri et al., 2022; Abdai and Miklósi, 2024). Analogously, animal robots used to investigate the social behavior of an animal species through animal-robot interactions can be called ethorobots.

A recent example of ethorobot is RoboFinch, a robotic zebra finch able to sing and display movements of the beak synchronized to the song, which can be used in both field and laboratory research to understand the effect of auditory and visual cues on songbird behavior (Simon et al., 2023). Intriguingly, ethorobots allow to study experimentally behaviors that are commonly expressed in nature but more difficult to induce in the laboratory. For example, male fiddler crabs are endowed with an enlarged sexually dimorphic claw which they wave at females during courtship rituals to capture their attention and to solicit female mate choice. Robotic fiddler crabs have been employed to understand the evolutionary adaptive value of this behavior and evaluate which specific characteristic of the waving behavior makes it attractive to females. In particular, Mowles and colleagues exposed female fiddler crabs to robotic crabs programmed to display different waving patterns, finding that the females strongly prefer a courtship featuring escalating waving, rather than constant waving or de-escalating waving (Mowles et al., 2018). Hence, as ethorobots are able to reproduce in the laboratory behaviors that are generally produced spontaneously in nature and are difficult or impossible to evoke and modulate in captive animals, these animal robots can be particularly useful tools for experimental ethology and ethological neuroscience, the branch of behavioral neuroscience focused on the investigation of the neural bases of natural species-specific behaviors (d’Isa and Gerlai, 2023). As can be seen from a recently published graph reporting the number of annual scientific articles on ethorobots published between 1998 and 2022, ethorobotics went through a progressive growth, with a sharp increase after 2010 (Siddall, 2023).

Rodents (mainly rats and mice) are the most common animal model in biomedical research (Makowska and Weary, 2021; Domínguez-Oliva et al., 2023). Coherently with the importance of these species for scientific research, robotic rats and mice have been attracting increasing efforts in bioinspired robotics.

Robotic engineer Robert Siddall recently underlined the potential usefulness of robotic rats for rodent behavioral research and reviewed the rat robots built up to now (Siddall, 2023). Similarly to the scientific literature on ethorobots, the number of scientific articles on robotic rats underwent a dramatic increase after 2010 (Siddall, 2023). Siddall summarized the technical features of 13 selected rat-like robots built between 2005 and 2022, such as Psikharpax (Meyer et al., 2005), iRat (Wiles et al., 2012), PiRat (Heath et al., 2018), NeRmo (Lucas et al., 2019), WR-7 (Yamada et al., 2021) and SQuRo (Shi et al., 2022). Moreover, Siddall proposed design considerations for new robotic rats that could be used as ethorobots (Siddall, 2023).

Mouse-inspired robots, on the other hand, have been created at least from the 1970s. In 1977 electronic engineer Donald Christiansen launched, from the Institute of Electrical and Electronics Engineers (IEEE) journal *Spectrum* (for which he was editor), the “Amazing MicroMouse Maze Contest,” challenging the readers to design and construct a robotic mouse capable of maze solving, and to compete in a contest that would have been held in 1979 in New York at the National Computer Conference (Christiansen, 2014). In a few years, micromouse competitions became a worldwide phenomenon. The first European micromouse context was held in 1980 in London, followed by Paris in 1981, while the first World Micromouse Contest was organized in Japan in 1985 (Christiansen, 2014). When *IEEE Spectrum* celebrated its 50 years anniversary in 2014, it was estimated that about 100 micromouse competitions were held annually worldwide (Christiansen, 2014). In 2024, the Annual MicroMouse Contest of the Applied Power Electronics Conference arrived to its 38th edition, while the All-Japan Micromouse Contest held its 44th edition. Research for the development of new models of mouse robots continues to be active. For instance, in 2023 NeRmo, an innovative robotic mouse with a flexible spine allowing improved locomotion, was created (Bing et al., 2023). In 2024 a similar flexible spine was implemented in a robotic rat, SQuRo-S, which exhibited stable walking on mud-sand, pipes and slopes of 20° (Wang et al., 2024).

The technological advancement of animal robots will make their employment for scientific research increasingly useful. However, clear experimental applications of animal robots should be identified in order to challenge engineers to design robots that can serve these experimental scopes. In the present work, we will describe possible practical applications of robotic animals for mouse behavioral testing across six different behavioral domains, namely courtship, parental care, antipredatory behavior, helping behavior, predation and territory defense-related aggression. Finally, in the conclusion we will consider the ethical and epistemological advantages of the use of robotic animals in behavioral neuroscience.

## 2 Robotic animals in mouse behavioral testing

### 2.1 Robotic birds of prey to study mouse antipredatory behavior in response to aerial threats

An innovative approach that is obtaining increasing attention in neuroscience research is testing the behavior of transponder-monitored rodents in the field (in the wild or in semi-natural outdoor enclosures), employing radio-frequency identification (RFID) systems to recognize the single free-ranging individuals (Vyssotski et al., 2002; Stradiotto et al., 2009; Daan et al., 2011; Spoelstra et al., 2016; Cope et al., 2019; Savage and Tremblay, 2019; Stryjek et al., 2021). Several behaviors that have already been studied in the laboratory could now be investigated also in the field, under naturalistic conditions, through these RFID technologies. An example is mouse antipredatory behavior in response to aerial threats.

Recent laboratory studies have shown that the presentation of an upper-field looming visual stimulus (an expanding black disc mimicking the approach of an aerial predator) elicits an innate flight-to-the-nest behavior in mice (Yang et al., 2020; Huang et al., 2020; Wang et al., 2023; Yuan et al., 2024). This defensive behavior in response to a visual threat could be studied also in the wild in a natural environment. However, the exposure of mice to birds of prey would be lethal for several individuals. A different strategy could be to release the rodents in a protected natural area without predators (a field enclosure) and to use bird-inspired drones. Indeed, the employment of robotic aerial predators, as for instance robotic eagles, could provide several advantages. On the one hand, time, frequency, duration and trajectory of the robotic eagle’s incursions would be totally under the control of the experimenter and could be manipulated as independent experimental variables. On the other hand, predator detection mechanisms and antipredatory behaviors could be easily studied in rodents in a natural environment without any risk that the experimental animals are killed or injured by a living predator. Interestingly, Robert Musters from the University of Groningen has recently developed RobotFalcon, a remotely-controlled robotic falcon, and with colleagues has successfully employed this robotic aerial predator to study the escape behavior of free-flying GPS-tagged homing pigeons in nature (Sankey et al., 2021), as well as of flocks of wild corvids, gulls, starlings and lapwings video-monitored through a ground camera (Storms et al., 2022, 2024).

### 2.2 Robotic rats to study mouse antipredatory behavior in response to terrestrial threats

Rats prey on mice and have a mouse-killing instinct (Albert et al., 1982, 1984, 1986). In the rat exposure test, a rat is used as stimulus animal and a mouse is allowed to interact with it (Yang et al., 2004; Wall et al., 2004; Perkins et al., 2009; Tovote et al., 2010; Campos et al., 2013; Li et al., 2019; Boillat et al., 2020). Predator fear in the rat-exposed mice is assessed by scoring avoidance, freezing or risk-assessment behaviors (such as stretched body posture and stretched approach). In free-interaction (unbarriered) protocols, for the safety of the mouse, the stimulus rat is anesthetized (Wall et al., 2004; Boillat et al., 2020).

However, this option still features risks for the stimulus animal. An option to avoid the risk of injuries is to use a wire partition between the mouse and the rat (Yang et al., 2004; Wall et al., 2004; Tovote et al., 2010; Campos et al., 2013; Li et al., 2019). Nevertheless, this protocol limits the possible interactions of the mice with the rat and consequently some variables, for example rat-contact time, are impossible to score. Alternatively, a dummy rat could be used, which would avoid the safety risks, but it appears that the dummy rats are far from being perceived as realistic by the mice, as dummy rat-exposed mice displayed significantly less defensive behaviors than towards a live anesthetized rat, namely less avoidance, stretched approach posture (a posture in which the body is stretched forward and the mouse remains motionless), stretched approaching (movement towards the rat with the body in a stretched position) and freezing (Yang et al., 2004).

Indeed, a robotic rat, capable of movements and vocalizations, would guarantee safety while offering a much more realistic option. Interestingly, some robots to test mouse defensive behaviors have already been designed. In 2014, Kuchiiwa and Kuchiiwa developed a novel semi-automated apparatus for the measurement of aggressive biting behavior in mice (Kuchiiwa and Kuchiiwa, 2014, 2016). This apparatus was endowed with computer-controlled spines that could move and touch the body of the mouse, inducing defensive biting in the mouse. Load sensors attached under the base of the spines could measure the number and the strength of the bites. Hence, the apparatus represents both a tactile stimulator and a biting response detector. A limit of this system is that it could be used to test only mice closed in a small box, with the spines emerging from the floor of the box. More recently, Zhang and colleagues have built a robotic terrestrial predator capable of a precise chasing movement with a high spatio-temporal resolution (Zhang et al., 2024). Notably, mice exposed to the approach of this robotic predator showed consistent escape behavior (Zhang et al., 2024). Another predator-like robotic chaser has been created by Pyeon and collaborators to investigate mouse defensive behaviors in response to a mobile terrestrial threat (Pyeon et al., 2025).

### 2.3 Robotic mice to study helping behaviors

Prosocial behavior is a voluntary action that benefits another individual (Jensen et al., 2014; Marshall-Pescini et al., 2016; Carballo et al., 2020). Prosocial behaviors include, for example, helping, sharing, donating, cooperating and comforting (Brief and Motowidlo, 1986; Wu and Hong, 2022). The underlying motivation of prosocial behaviors can be classified as selfish, mutualistic or altruistic, based on whether the final aim of the behavior is to benefit, respectively, oneself, both oneself and the other, or just the other. On the other hand, the social interaction deriving from a prosocial behavior can be of only two types: mutualistic or altruistic. This is due to the fact that even a prosocial behavior emitted with a selfish motivation still leads to a benefit for the receiver, by definition. Social interactions deriving by prosocial behaviors are opposed to social interactions that are detrimental for the receiver, such as predatory or parasitic interactions. A clear example of selfish prosocial behavior is provided by the cleaner fish *Labroides dimidiatus*, that inspects the surface and gills of a so-called client fish removing ectoparasites, scales and dead tissue (Soares, 2017). While the cleaner fish removes these items to eat them and feed itself (a selfish motivation), the removal of the parasites and of dead tissues is a benefit for the client, which makes this social relationship mutualistic.

Several behavioral protocols have been used to study rodent prosocial behaviors. Considering, for instance, helping behavior (providing aid to an individual in condition of need), rats have been found to help conspecifics that were in difficulty under several different circumstances. In a seminal work from 1962, Rice and Gainer showed that rats press a bar to lower a conspecific suspended in the air through a hoist (Rice and Gainer, 1962). Moreover, rats open the door of a plastic restraint box to free a conspecific that is trapped inside (Ben-Ami Bartal et al., 2011, 2014, 2016; Blystad et al., 2019; Mason, 2021). Interestingly, rats opened the box significantly less frequently when the box was empty or contained an object (Ben-Ami Bartal et al., 2011). Additionally, through the same restraint box behavioral protocol, researchers showed that rats opened the door for both a familiar cage-mate and a stranger rat (Ben-Ami Bartal et al., 2014). On the other hand, they did not open the door for a rat of a different strain unless they had previously lived together with it in the same home-cage (Ben-Ami Bartal et al., 2014). Finally, in a third type of behavioral protocol, rats freed a soaked cage-mate trapped in an area flooded with water, by opening a door allowing the soaked rat to reach a safe dry area (Sato et al., 2015).

While it is clear that rats help conspecifics, the motivations of these behaviors are less evident. In their seminal article presenting the suspended rat experiment, Rice and Gainer suggested that the behavior of the helper rat is altruistic (Rice and Gainer, 1962). Nevertheless, the following year, Lavery and Foley criticized this interpretation and performed a series of experiments without a distressed rat, showing that rats press the bar to lower the suspended object even if it is a sound box reproducing pre-recorded rat vocalizations or white noise (Lavery and Foley, 1963). Lavery and Foley suggested that the helper rat was not altruistic, but rather was pressing the bar only to stop an aversive acoustic stimulation, which would actually make its motivation selfish. Although the fact that rats consider aversive the distress vocalizations of another rat is not actually incompatible with the rats lowering a conspecific for altruistic reasons, Lavery and Foley’s results may cast doubts on the motivations of the helper rats. In order to distinguish selfish helping behavior from truly altruistic helping behaviors, specific experimental protocols can be designed. In particular, if the helper rat still performs the helping behavior even when helping a conspecific leads to a disadvantage for itself, then the helping behavior can be classified as truly altruistic. Notably, evidence from costly help tests showed that rats chose the altruistic option and help their conspecific accepting a disadvantage for themselves. For example, in the soaked conspecific protocol, when, after the help test, the rats were exposed to a two-choice test in which two doors were present, one giving access to a chamber with a highly palatable food (chocolate cereals) and one allowing their soaked cage-mate to escape, the rats’s first choice was to free their cage-mate in the vast majority (~80%) of the trials (Sato et al., 2015). This finding suggests that the helping behavior of the rats towards the soaked conspecific is altruistic and motivated by empathy for the conspecific in difficulty.

Helping behaviors have been observed also in mice, although up to now they have been studied less extensively than in rats. For instance, in the restrained conspecific paradigm, mice freed the conspecific by opening the lid of the tube in which the conspecific was restrained (Ueno et al., 2019). In future studies, it would be interesting to investigate further the mouse helping behaviors and their underlying motivations.

Unfortunately, in all the three aforementioned behavioral paradigms for the study of helping behavior (suspended conspecific, restrained conspecific and soaked conspecific), in order to test the subject rat/mouse, a stimulus rat/mouse must be kept in condition of distress. The employment of a robotic rodent as stimulus could be particularly useful to avoid this distress. Indeed, it has already been shown that rats help robotic rats (Quinn et al., 2018).

Interestingly, vocalizations appear to have a great importance for cooperative behaviors in rodents. For instance, in an experiment in which pairs of familiar rats had to learn to nose-poke simultaneously some holes to obtain a sucrose reward, the rate of cooperation progressively increased over the course of 44 days of training, in parallel with an increase of the 50 KHz vocalizations (Łopuch and Popik, 2011). Notably, when vocal communications were impeded through the insertion of a partition between the rats, the success rate of the pair dropped. On the other hand, if the rats were separated by a wire mesh partition that allowed to hear each other, the cooperative success was re-established, showing that physical contact was not required for cooperation, but exchange of ultrasonic vocalizations was. Robotic rats capable of realistic rat-like movements and placed in specific situations of danger (for example a suspended robotic rat struggling in the air with the paws) could be used to reproduce, at time-points and for durations decided by the experimenter, vocalization playbacks or even experimentally manipulated audios, allowing the adoption of experimental designs that could explore in unprecedented ways the role of vocal signalling in inducing helping behaviors in rats. Smaller-sized robotic mice could analogously be used to study helping behaviors in mice.

### 2.4 Robotic mice to study mouse defensive territorial behavior

Mice are territorial animals. Consequently, if they are housed in a home-cage, they consider it their territory and, in the case of an intrusion by another mouse, they tend to defend the territory by attacking the intruder. Upon this simple defensive reaction is based the most common behavioral test of aggression for mice: the resident-intruder test (Koolhaas et al., 2013; Ruzza et al., 2015). In this test, an unfamiliar mouse (the intruder) is introduced in the home-cage of a resident mouse and the resident’s behavioral responses to the intrusion are scored. The resident is the subject animal, while the intruder is used as stimulus animal.

Unfortunately, this test features a risk of physical attack and consequently injury for the intruder (Koolhaas et al., 2013), raising ethical concerns related to animal welfare (d’Isa and Gerlai, 2023). The employment of a robotic mouse as stimulus animal would completely avoid such risks. Moreover, it would make possible to manipulate certain behaviors of the intruder, such as posture or ultrasonic vocalizations, and test if the aggressive behavior of the resident is reduced or increased.

Robotic mice could also be used in other social behavior tests, such as the social interaction test, in which a subject mouse is exposed to an unfamiliar stimulus mouse in a neutral context (not the home-cage of one of the two mice) and the social behaviors of the subject mouse are assessed. In this test, in order to avoid fights and possible injuries, barriers (made of transparent plexiglass or wire mesh) are often used to keep separated the mice (for instance: Kaidanovich-Beilin et al., 2011; Papale et al., 2017; Harda et al., 2022; Kurahashi et al., 2024). However, this option does not allow physical contact and interaction with the stranger, which are important components of sociability. The employment of robotic mice as stimulus animals would guarantee the possibility to score contact time and other variables related to physical interaction, such as nose-to-nose sniffing or allogrooming (i.e., mouthing and licking the fur of another mouse to clean it), in complete safety.

### 2.5 Robotic crickets to study mouse predatory behavior

Mice are commonly considered prey animals. Nevertheless, mice are also predators. Indeed, insect hunting is common in several rodents, including mice (Galvin et al., 2021). In a laboratory setting, when mice are presented with a cricket, they rapidly attack and kill it. At the end of the 1960s, the comparative psychologist Karla Thomas Butler^1^, from California State University, was the first to study mouse predatory behavior in the laboratory (Thomas, 1969). In the early 1970s, Karla Thomas Butler exposed seven strains of laboratory mice to crickets and found that all the mouse strains showed cricket-killing behavior, underlining the usefulness of this behavior for laboratory behavioral tests (Butler, 1973). A few years later, the reliability of cricket-killing in mice was confirmed and mouse cricket-killing was defined as “an inexpensive, easily obtainable model of predation” (Lowe and O’Boyle, 1976). Indeed, Butler’s cricket-attacking test became the main test to study mouse predatory behavior (Lowe and O'Boyle, 1976; Nesbitt et al., 1979; Kantak et al., 1980; Hahn et al., 1991; Sandnabba, 1995; Gammie et al., 2003; Vishnivetskaya et al., 2007; Cai et al., 2020; Galvin et al., 2021; Johnson et al., 2021; Groves Kuhnle et al., 2022). The cricket-attacking response is instinctual and is present also in laboratory mice that have never seen before a cricket in their whole lifetime. However, learning may perfect this behavior, as shown by the decrease of hunting times in mice exposed to crickets over the course of multiple days (Galvin et al., 2021).

Through robotic crickets, several parameters of the stimulus animal (such as the posture, the latency to hop, the speed of hopping and the number of hops) could be controlled by the experimenter. Moreover, no cricket would be killed during the experiments and mice would not risk to get harmed by cricket bites during the fight. Interestingly, simple prey-like robots (Hexbug Nano toys, which resemble cockroaches) have already been tested with mice and were successful in eliciting hunting behaviors in mice which were optogenetically stimulated in a subset of GABAergic neurons within the lateral hypothalamus, an area involved in the control of predatory behavior (Rossier et al., 2021).

### 2.6 Robotic mouse pups to study parental care

Mice are altricial, meaning that at birth they are highly immature and totally dependent on parental care for survival. Mouse newborns are blind, deaf and without fur (Latham and Mason, 2004). Their motor abilities are very limited, which makes them unable to stand on their four paws and walk (Feather-Schussler and Ferguson, 2016). They are ectothermic (without an external source of heat their body temperature rapidly drops) and their thermoregulation depends on maternal care, both directly (body contact) and indirectly (quality of the nest built by the mother) (Brust et al., 2015). For food, newborns analogously rely on the mothers, that nourish them through nursing. Even for digestion, newborn pups are dependent on maternal care and they emit specific ultrasonic vocalizations, called wriggling calls, to demand from the mother licking of the abdomen that stimulates digestion and defecation (Ehret and Bernecker, 1986).

In a laboratory setting, if a pup is collected from the nest and released in a different part of the cage, the mother will readily reach the pup, pick it up with the mouth and carry it back to safety in the nest. This behavior is known as pup retrieval. Through the so-called pup retrieval test (PRT), parental care can be assessed in mice by measuring variables such as latency to retrieve the first pup, latency to retrieve a pup after a previous retrieval, number of pups retrieved and total time spent retrieving pups.

Different types of pup vocalizations can specifically modulate maternal care behaviors. While low-frequency wriggling calls (under 10 kHz) trigger maternal licking and nest-building (Ehret and Bernecker, 1986), the brief and higher frequency ultrasonic vocalizations (50–80 kHz) of isolated pups trigger retrieval (Hofer et al., 2002).

Although in refined versions of the PRT, the pups are kept warm by using heat pads or previously heated supports (Lee et al., 2021; Winters et al., 2022, 2023), through the employment of robots, the induction of psychological stress deriving from isolation could be completely avoided for the pups. Robotic pups could be used to evaluate parental care behavior in different contexts and environments. Since pups are hairless, it would be easy to reproduce realistic robotic pups with rubber skin and, since the motor abilities of pups are scarce, it would be easy to implement such simple limb and head movements. Importantly, robotic pups could emit specific acoustic signals mimicking pup vocalizations, in order to induce retrieval behavior in the dams. The number, acoustic intensity, acoustic frequency and type of the pup calls would be under the total control of the experimenter, and the different effect of each of these parameters on retrieval behavior could be easily dissected.

### 2.7 Robotic female mice to study male courtship and mating behavior

Female mice have an active role in initiating sexual interactions with males. When female mice encounter a male that meets their preferences, they signal their sexual interest by displaying a series of proceptive behaviors, i.e., behaviors that encourage sexual approach by the intended receiver (Beach, 1976). Proceptive behaviors of female mice include hopping, darting movements and ear-wiggling, which have a sexually arousing effect on males (Latham and Mason, 2004). Male mice attracted by female mice approach them and start a sequence of ultrasonic vocalizations that has been defined as courtship song, due to its subdivision in syllable types which are arranged nonrandomly and repeated according to a specific temporal structure (Holy and Guo, 2005). Interestingly, courtship songs contain syntactical features that are strain-specific (Melotti et al., 2021), but are also characterized by individual signatures (Marconi et al., 2020; Melotti et al., 2021). Female mice prefer vocalizing males over non-vocalizing males (Pomerantz et al., 1983) and, within vocalizations, they show a preference for songs with certain syntactical structures, in particular the more complex and elaborate songs (Chabout et al., 2015). Through the darting movements, females perform zig-zag runs that solicit chasing by males. Chasing is an important part of mouse courtship (Van Oortmerssen, 1971). During this courtship chase, male vocalize and females vocalize in response. Females that choose to vocalize during the chase slow down their running speed, allowing the males to catch them more easily, indicating that this female singing back is another proceptive behavior (Neunuebel et al., 2015). Finally, if courtship is positively received by female mice, they may accept physical contact with the male and indicate their willingness to copulation through receptive behaviors, in particular by adopting a typical lordosis posture, an arching of the back that facilitates mounting by males (Hellier et al., 2018).

During normal sociosexual communication between opposite-sex mice, it is not possible to separate the single elements of the female proceptive behavior nor to decide which of them will be predominantly displayed. On the other hand, by using robotic female mice, specific patterns of proceptive display could be pre-programmed. This would bring several advantages. First, the relative attractiveness to males of the single elements of female proceptive behavior could be studied, including single postural, locomotor and acoustic cues of the multimodal female intersexual signalling pattern. For instance, it would be possible to understand if single elements alone stimulate courtship singing in males or which of the single elements is more effective, leading to a higher probability and/or a higher intensity of male courtship. Additionally, each sensory cue could be modulated to understand the differential effects of its variation on male behavior (for example, in the case of acoustic signalling, low-frequency broadband female vocalizations could be compared with ultrasonic female vocalizations). Finally, it would be possible to test combinations of multimodal cues, also in different temporal sequences, to understand which sensory stimuli may have additive effects or even synergic effects in promoting courtship in male mice.

## 3 Conclusion

Robotic animals may benefit behavioral neuroscience research with both ethical and epistemological advantages. From an ethical point of view, the employment of ethorobots as stimulus animals in social tests which feature a risk of aggression for the stimulus animal (such as the resident-intruder test) would increase the animal welfare in the experimental practice. In terms of the 3Rs, this would be a refinement of the current behavioral testing procedure. Furthermore, if ethorobots are used as stimulus animals in such risk-featuring protocols, in addition to optimizing safety, less living animals would be employed in the experiments, in accordance with the reduction principle.

From the 17th century, society has been more and more recognizing the responsibility of humans towards the welfare of animals under human care (for a history of early legislations and literature on animal welfare, see section 5.1 of d’Isa and Abramson, 2025). Interestingly, in rodent behavioral science, care for the welfare of the studied subject was present from the very first rodent behavioral study in 1822 (d’Isa, 2025). Significantly, over the past 40 years, the scientific community has been devoting a progressively increasing attention to animal welfare issues, as can be seen by an analysis of the number of articles in the biomedical archive PubMed that mention the phrase “animal welfare” between 1980 and 2023 (d’Isa et al., 2024). Up to now, several options have been proposed for an animal-friendly behavioral testing that does not harm nor stress the animals (d’Isa and Gerlai, 2023; d’Isa et al., 2024). Examples are the object recognition test (d’Isa et al., 2014), the spontaneous alternation T-maze (d’Isa et al., 2021a) and the hole-board test (d’Isa et al., 2021b). New options are continuously being explored, especially as new technologies become available. For future behavioral research, robotic animals could be one of the most promising new frontiers of animal welfare optimization.

Moreover, it is important to note that the use of robotic animals in behavioral sciences can bring not only ethical advantages (in terms of both refinement and reduction), but also epistemological/methodological advantages. Indeed, if sufficiently realistic robotic animals are designed, this would allow a level of investigation that in behavioral experimental designs with only living animals cannot be achieved. If, for instance, the reactions to aggressive displays or to courtship displays are studied, in laboratory settings the study typically involves a stimulus animal and a subject animal. When both the stimulus animal and the subject animal are living organisms, the behavior of the stimulus animal remains, at least in part unpredictable. Additionally, if the aggressive or courtship display features, for example, a collection of 4–5 behaviors, these behaviors cannot be separated. On the other hand, by using a robotic animal as stimulus animal, unprecedented possibilities are offered to the experimenter. The behavior of the robot can be fully under the control of the experimenter and specific behaviors may be emitted at will without temporal constraints. Furthermore, the single behaviors of the display (such as change of posture, vocalization or a specific motor pattern) may be reproduced independently, allowing to dissect the effect of each behavioral component on the social behavior of the subject animal. This enables to understand which of these components are sufficient to obtain a target behavioral response of the subject animal, and which components, on the other side, are both sufficient and necessary. In particular, a behavioral component that is sufficient but not necessary would be able to elicit the target behavioral response in the subject animal, but the same behavioral response could be obtained also by one or more of the other behavioral components of the display. On the other hand, a behavioral component that is both sufficient and necessary could not be substituted by any of the other components to generate the target effect. Analogously, by employing adequately designed robots, it would also be possible to dissect the specific effect of single behavioral components of the display of the stimulus animal on the physiological responses of the subject animals, for instance changes in stress hormones, sexual hormones, neurotransmitters or neuropeptides.

A possible limitation of the use of robotic animals with living rodents is the realism of the ethorobots. However, it is important to underline that the zoorobots do not need to be perfect replicas of the animals they imitate, but just models sufficiently realistic to elicit the target behavioral responses in the tested living animals. Moreover, the realism of the robot should be evaluated from the sensorial perspective of the tested living animal. For instance, the main sense of rodents is olfaction. Hence, as has already been proposed by others (Siddall, 2023), olfactory stimuli could be applied to the ethorobots to enhance their realism to rodents. Depending on the experimental paradigm, different scents could be employed. In social interaction tests, the bedding of an unfamiliar mouse could be used to leave its scent on a robotic mouse before presenting the ethorobot to the tested mouse. On the other hand, in tests of antipredatory behavior, the odor of natural predators of rodents (such as coyotes, foxes and cats) could be applied to a chasing ethorobot to make it more salient.

**Figure 1:**
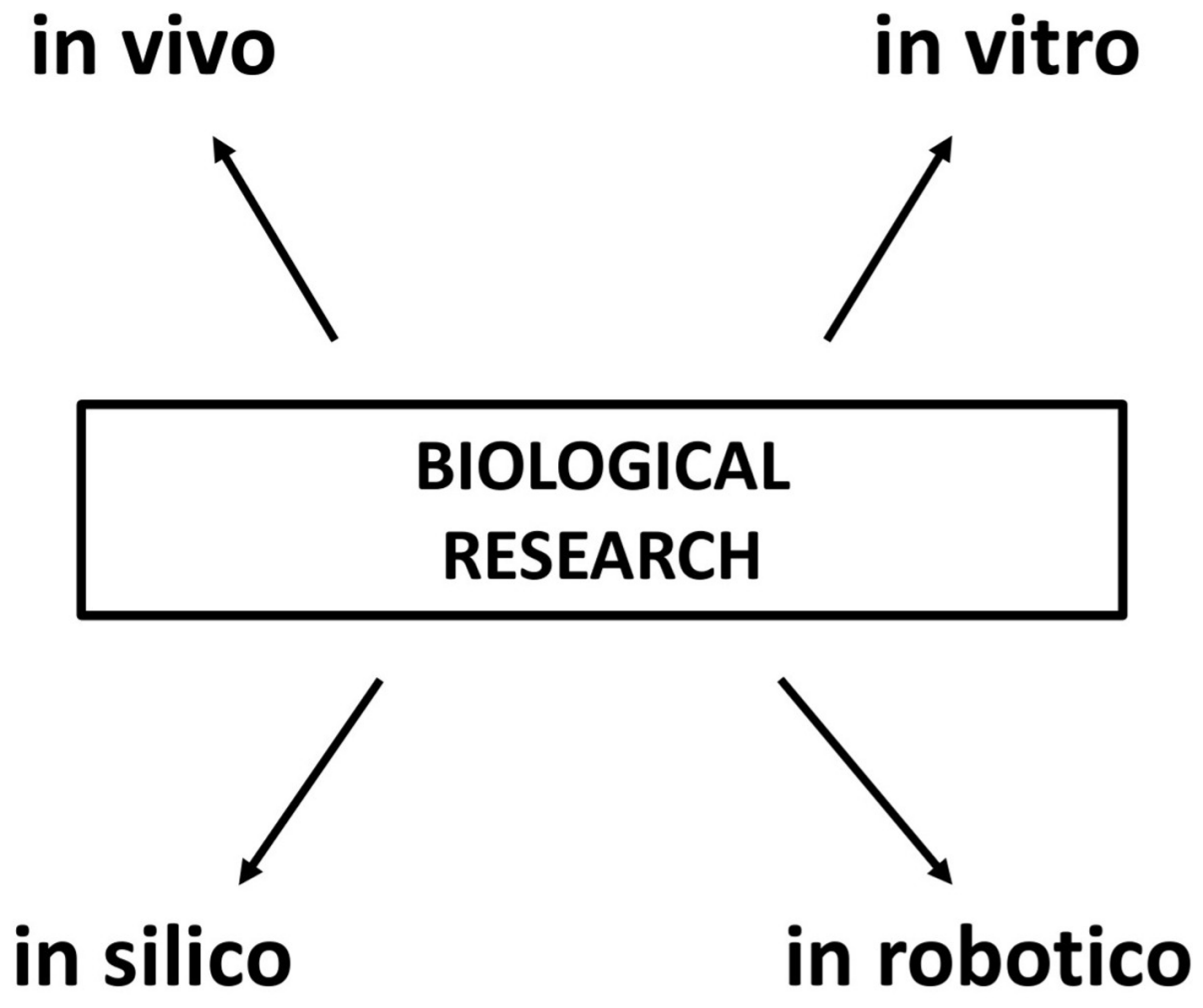
Four major approaches in biological research. Biological hypotheses can be tested through experiments *in vivo* (with complete living organisms), *in vitro* (with living cells that belonged to a more complex organism), *in silico* (with a computer simulation) and *in robotico* (with robotic models as subjects).

In the present article, we considered how robotic animals could benefit behavioral research as stimulus animals. However, robotic animals could also be used as subject animals (Datteri et al., 2022). In these cases, the animal robot would represent an experimental model of the living animal. Soft robots endowed with artificial intelligence would be of particular usefulness to implement this approach (Abramson and Levin, 2021). It would be possible to study the behavior of a single robotic animal alone or groups of animal robots could be used to study collective behavior (Garnier, 2011; Brambilla et al., 2013; Schranz et al., 2020). By employing robotic animals as subject animals, a new type of experimental testing would be available for biological sciences. Indeed. as schematized in Figure 1, it would be possible to test scientific hypotheses not only *in vitro* (with living cells that belonged to a more complex organism), *in vivo* (with complete living organisms) and *in silico* (with a computer simulation), but also *in robotico* (with robotic models as subjects). Behavioral testing *in robotico* can be particularly useful to help to understand how, in living animals, complex social behaviors can emerge from simple behaviors of freely interacting individuals. For instance, rat pups show a tendency to aggregate in compact groups, an adaptive behavior that prevents heat dispersal and makes the offspring more easily monitorable by the mother. It was previously believed that this aggregation behavior was due to an instinctual attraction of pups towards objects and other pups. However, by programming rat-like robots to move randomly in an arena, without the influence of any sensor, May and colleagues found that exactly the same aggregating behavior as pup rats placed in the arena could be obtained by the robots, indicating that rat pup aggregation could actually be explained simply by body shape, friction and random movement (May et al., 2006). In the future, an alternation between animal–animal experiments and animal-robot experiments could bring behavioral research to an optimized process of scientific discovery based on observation in living animals, simplification, robotic modelling, hypothesis formulation, hypothesis testing in living animals and synthesis (Alberts, 2012).

Three of the currently most promising trends in behavioral neuroscience are home-cage behavioral monitoring systems (Mingrone et al., 2020; Voikar and Gaburro, 2020; d’Isa and Gerlai, 2023; Kahnau et al., 2023; Lipp et al., 2024), seminatural environments (Hernández-Arteaga and Ågmo, 2023; Shemesh and Chen, 2023) and animal robots (Abdai et al., 2018; Romano and Stefanini, 2021; Landgraf et al., 2021; Datteri et al., 2022; Romano et al., 2024). An interesting possibility for the future would be to combine these three approaches, creating an ample space for animal housing which: (1) reproduces the elements of a natural environment; (2) contains the interactive elements and the automated behavior recording systems of home-cage monitoring systems; (3) can host also robotic animals for the study of complex social behaviors of the housed species. Importantly, each of these three approaches (automated home-cage monitoring, seminatural environment and animal robots) leads to advantages in terms of animal welfare. Indeed, by housing experimental animals in this naturally inspired and highly technological habitat, it would be possible to maximize both the welfare for the housed species and the scientific opportunities for the researchers studying them.

## Data Availability

The original contributions presented in the study are included in the article/supplementary material, further inquiries can be directed to the corresponding author.

## Footnotes

^1^Known as Karla Thomas before her marriage and as Karla Butler afterwards.
